# Preparation and Evaluation of Oseltamivir Molecularly Imprinted Polymer Silica Gel as Liquid Chromatography Stationary Phase

**DOI:** 10.3390/molecules23081881

**Published:** 2018-07-27

**Authors:** Ya-Jun Yang, Xi-Wang Liu, Xiao-Jun Kong, Zhe Qin, Zeng-Hua Jiao, Shi-Hong Li, Jian-Yong Li

**Affiliations:** Key Laboratory of New Animal Drug Project of Gansu Province; Key Laboratory of Veterinary Pharmaceutical Development, Ministry of Agriculture; Lanzhou Institute of Husbandry and Pharmaceutical Sciences of CAAS, No. 335, Jiangouyan, Qilihe district, Lanzhou, Gansu province, Lanzhou 730050, China; yangyue10224@163.com (Y.-J.Y.); xiwangliu@126.com (X.-W.L.); kongxj009@163.com (X.-J.K.); qinzhe@caas.cn (Z.Q.); jiaozengh@163.com (Z.-H.J.); lzlishihong@163.com (S.-H.L.)

**Keywords:** oseltamivir, molecularly imprinted polymer (MIP), silica gel, liquid chromatography stationary phase, peramivir

## Abstract

To improve the chromatographic performance of an oseltamivir (OS) molecularly imprinted polymer (MIP), silica gel coated with an MIP layer for OS (OSMIP@silica gel) was prepared by the surface molecular imprinting technology on the supporter of porous silica gel microspheres. A nonimprinted polymer with the silica gel (NIP@silica gel) was also prepared for comparison. The obtained particles were characterized through FT–IR, scanning electron microscopy, specific surface area analysis, and porosity measurements. The results indicated that the polymer was successfully synthesized and revealed the structural differences between imprinted and nonimprinted polymers. The results of static adsorption experiments showed that adsorption quantity of the OSMIP@silica gel for OS was higher than that for NIP@silica gel, and the OSMIP@silica gel had two kinds of affinity sites for OS but the NIP@silica gel had one. The chromatographic performance of the OSMIP@silica gel column had significant improvement. The imprinting factor of the OSMIP@silica gel column for OS was 1.64. Furthermore, the OSMIP@silica gel column showed good affinity and selectivity for template OS and another neuraminidase inhibitor, peramivir, but not for quinocetone. These results indicated that the prepared OSMIP could be used to simulate the activity center of neuraminidase, and the OSMIP@silica gel column could be also employed in future studies to search for more active neuraminidase inhibitor analogues from traditional Chinese herbs.

## 1. Introduction

Molecular imprinting technology (MIT), which simulates the specific interaction between enzyme and substrate, is employed to prepare polymers with recognition sites of predetermined specificity to target compounds or template molecules. The perfect selectivity, high binding affinity and physical robustness of molecularly imprinted polymers (MIPs) enable them to be surrogates for native receptors in drug discovery [1,2,3,4]. The structures of affinitive compounds, which were trapped by MIPs from matrixes, had similarity to the template. These affinitive compounds would have similar bioactivity to the template from a structure–activity relationship point of view. Nowadays, MIPs are used for screening analogues with similar bioactivities to search for new drug candidates from plant extracts [5,6] or combinatorial chemistry libraries [7]. The aim of our serial studies on MIP was to develop a nonbiological method for screening active components from traditional Chinese medicine (TCM) by coupling a liquid-chromatography (LC) column with MIPs [8,9].

In our previous studies [8,9], the influenza virus neuraminidase inhibitor oseltamivir (OS) was used as template to synthesize an oseltamivir molecularly imprinted polymer (OSMIP) through bulk polymerization. The bulk polymers were grinded, sieved and packed into a stainless-steel LC column (150 mm × 4.6 mm, 45–60 μm). The specific affinity tests of OSMIP for the template molecule OS and other different compounds, such as chlorogenic acid, phillyrin and aspirin, have been investigated. The results showed that OSMIP had a very high affinity and selectivity to separate the template molecule OS from other compounds. The LC column was employed with LC–MS, and an affinitive component with *m*/*z* 249, matrine, was found from the chloroform extract of a TCM liquid preparation. Matrine had similar bioactivities to OS against avian influenza virus H9N2 in vitro, both alleviating cytopathic effect and hemagglutination inhibition, which proved that the availability of MIP worked as a nonbiological method for screening active components from TCM against influenza virus.

Shoravi S et al. also reported a preparation method of OSMIP [10]. The full-system molecular dynamics-based studies were used to screen the molecular imprinting prepolymerization systems of OSMIP. The results showed that the prepared OSMIP had the highest affinity for OS, in agreement with the predictions made from the simulations, indicating that the method was an effective tool in MIP design.

However, conventional methods for preparation of MIP monoliths include either the time-consuming grinding/sieving steps that lead to irregular particles with poor mechanical and chromatographic performance or use of complicated polymerisation systems in order to get polymer beads. The prepared OSMIP particles are amorphous too [8], so the background pressure of the LC column with OSMIP was very high. MeOH–MeCN–formic acid (75:25:0.01) was used as the mobile phase at the flow rate of 0.075 mL·min^−1^ to ensure the system pressure was lower than the packing pressure of 2000 psi. Therefore, the peak widths of OS and other analytes were very wide and the analytical time of every sample was longer than 5 h [8,9]. On account of this, one of the main objectives of this study was to improve the chromatographic performance of the OSMIP LC column and shorten the analytical time of the samples.

To overcome these problems, surface MIT has been developed using silica gel as supporter [11,12,13,14,15]. Silica gel particles, because of their high stability, chemical inertness and nonswelling property, have been employed to be a proper material for surface molecular imprinting. The specific recognition sites are embedded on the surface of the polymer, which provides high specificity and selectivity for the target molecule and its analogues. Therefore, the disadvantages of traditional MIPs have been avoided to some extent [12,16,17].

In this paper, OSMIP was synthesized on the surface of porous silica gel microspheres (OSMIP@silica gel) and characterized. Adsorption properties of the OSMIP@silica gel were evaluated by static adsorption experiments. The chromatographic performance of the OSMIP@silica gel has been improved significantly compared to our previous reports [8,9]. The column had good affinity and selectivity for OS and peramivir, which is a neuraminidase inhibitor too. This study will provide the basis for further screening of more active analogues of neuraminidase inhibitors from traditional Chinese herbs or other compound libraries. Moreover, the nonbiological method for screening active components from TCM by coupling an LC column with MIPs might be promoted through the present and subsequent studies.

## 2. Results and Discussion

### 2.1. Derivatization, Polymer Synthesis and Characterization

Silica gel as a solid supporter is of great importance because it possesses some definite advantages, such as it is nondeforming, has good mechanical strength and is heat stable [16,18]. Besides, the surface functionalization of silica gel to introduce additional functionality can be easily obtained by organosilicone [16].

To remove impurity and increase the quantity of hydroxyls on the surface, the silica gel was processed according to the reported method [19]. Silica gel was chemically modified with 3-(trimethoxysilyl)propyl methacrylate (KH570) to introduce double bonds on the surface mainly according to the reported method [19]. The process of imprinting synthesis at the surface of silica gel was mainly according to our previous study and also referenced the reported methods [8,17,20]. The prepolymerization was first done at 50 °C for 6 h, and the final polymerization was completed at 60 °C for 12 h. The particles were further aged at 85 °C for 6 h for obtaining a high crosslinking density [20].

**Figure 1:**
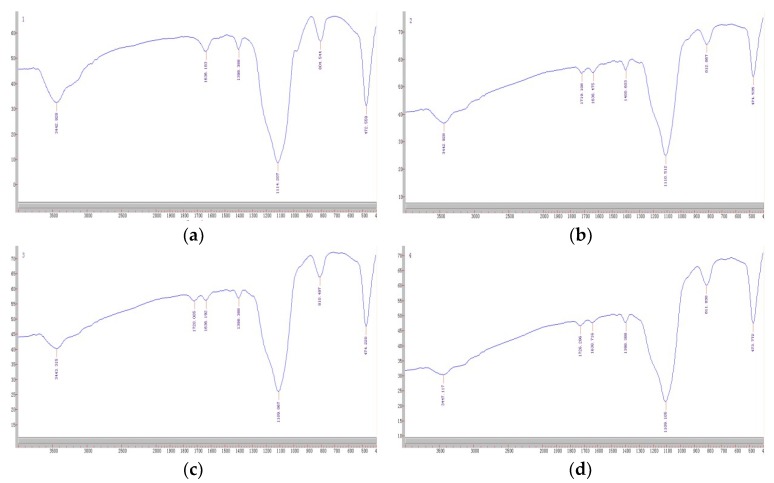
FT–IR spectra of activated silica gel (**a**), KH570@silica gel (**b**), MIP@silica gel (**c**), NIP@silica gel (**d**).

Figure 1 shows the FT–IR spectra of activated silica gel, KH570@silica gel, OSMIP@silica gel and nonimprinted polymer with the silica gel (NIP@silica gel). The main absorption bands at around 3440 cm^−1^, 1630 cm^−1^ and 1110 cm^−1^ were mainly assigned to the stretching vibrations for Si–O–H of silica gel, and the bands around 810 and 470 cm^−1^ were attributed to Si–O vibrations [18,21]. The absorption bands at around 1720 cm^−1^ on Figure 1b–d were attributed to C=O stretching vibration and confirmed that the silica gels were modified with KH570 and coated with the applied crosslinker. In addition, it could be clearly seen that there were no differences among the spectra of the KH570@silica gel, OSMIP@silica gel and NIP@silica gel. In this case, the presented spectra showed very similar positions, appearances and intensity of the major bands, which confirmed the fact that they had similar backbones.

**Figure 2:**
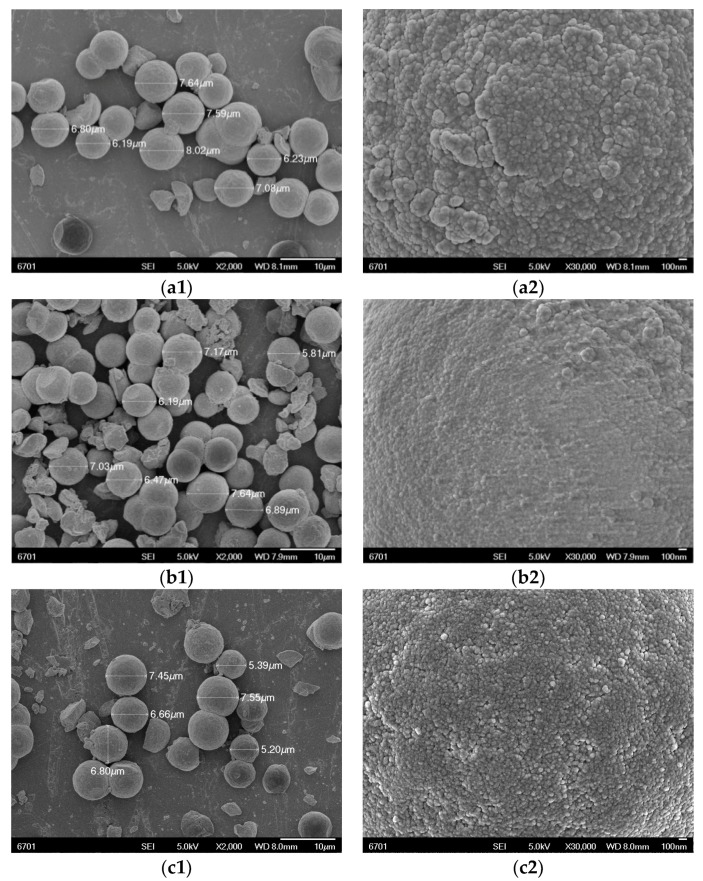
SEM images. OSMIP@silica gel (**a2**) with the roughest surface. NIP@silica gel (**b2**) with smoother surface. KH570@silica gel (**c2**) with rougher surface than NIP@silica gel.

The SEM images of the OSMIP@silica gel, NIP@silica gel and KH570@silica gel are shown in Figure 2. OSMIP@silica gel, NIP@silica gel and KH570@silica gel were spherical particles with diameters between 6 and 8 μm. The SEM images clearly showed that there were substantial differences on the surface of the OSMIP@silica gel compared to the NIP@silica gel and KH570@silica gel. The OSMIP@silica gel had the roughest surface at the same magnification (×30,000). The surface of the KH570@silica gel was also rougher than the NIP@silica gel. The compact NIP coating was the possible reason that the NIP@silica gel had a smoother surface than other particles.

As we know, porous silica gel microspheres have huge surface areas. In the present report, the labeled surface area was 400 m^2^ g^−1^. Hence, the surface areas of silica gels were employed as a platform for the surface imprinting process. The surface areas and porosity of the OSMIP@silica gel and NIP@silica gel were measured by nitrogen adsorption porosimetry. Specific surface areas were calculated using the Brunauer, Emmett and Teller (BET) method, and the specific pore volumes and average pore diameters were calculated by the BJH method, and the results are shown in Table 1.

In comparison to silica gel, corresponding decreases in specific surface areas, average pore diameters and pore volumes of OSMIP@silica gel and NIP@silica gel were observed. The particle diameters of OSMIP@silica gel and NIP@silica gel were increased compared to silica gel, resulting in a smaller surface area with an average pore diameter and pore volume. The NIP@silica gel particles showed larger porosity parameters than the OSMIP@silica gel. However, the obtained results indicated that there were no significance differences of these parameters between OSMIP@silica gel and NIP@silica gel. It seems that the addition of OS as a template molecule into the polymerization process generates the tiny difference. These changes have demonstrated that the OSMIP layer was formed on the surface of the silica gel successfully.

### 2.2. Adsorption Experiments

**Figure 3:**
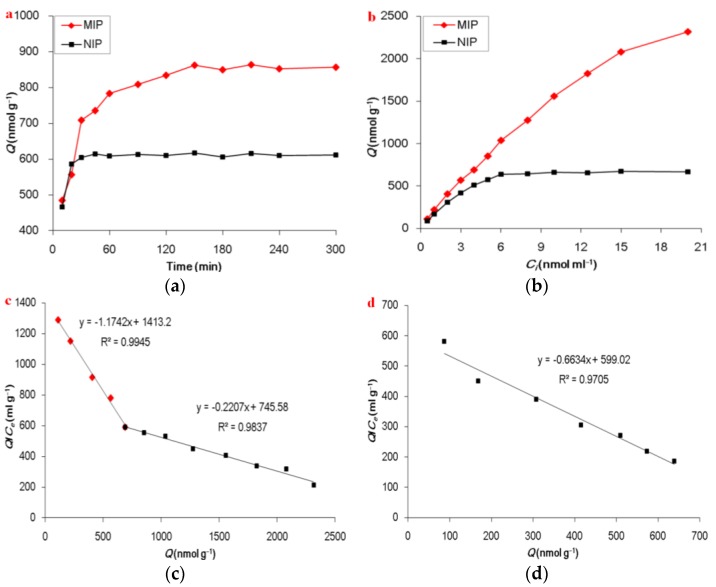
Kinetic adsorption curves of OS on OSMIP@silica gel and NIP@silica gel (**a**), static adsorption of OS on OSMIP@silica gel and NIP@silica gel (**b**), Scatchard plot for OS on OSMIP@silica gel (**c**) and NIP@silica gel (**d**).

The kinetic adsorption is one of the most important factors in evaluating the adsorption efficiency. The effects of the adsorption time of the OSMIP@silica gel and NIP@silica gel for the OS solution were investigated by varying the time at a range from 10 to 300 min [10,22]. The kinetics adsorption curves are shown in Figure 3a. It shows a significance increase in adsorption capacity of the OSMIP@silica gel vs. the NIP@silica gel. The results indicated that the adsorption amounts of the OSMIP@silica gel increased rapidly within 150 min and then reached equilibrium gradually, compared with the nearly saturated adsorption of NIP@silica gel after just 30 min.

In order to further study the binding properties of the OSMIP@silica gel, Scatchard analysis was performed. In general, the Scatchard plot is used for the evaluation of adsorption parameters [23]. Furthermore, the Scatchard plot can indicate how many kinds of binding sites exist in the MIPs. If the plots can be fitted into one line, it indicates that there is only one kind of binding site existing in imprinted cavities. If it can be fitted into more than one line, there may be several kinds of binding sites existing. Scatchard analysis was calculated through the Scatchard equation [22]. The calculated equation was exhibited as the following:

(1) $$\frac{Q}{C_{e}}=-\frac{Q}{K_{D}}+\frac{Q_{\max}}{K_{D}}\;$$

where *Q* (nmol·g^−1^) is the amount of OS bound to the OSMIP@silica gel at equilibrium, *C*_e_ (nmol·mL^−1^) is the free OS concentration in matrix solution, *Q*_max_ (nmol·g^−1^) is the maximum binding capacity, and *K*_D_ is the dissociation constant. 

It is possible to estimate the *Q*_max_ and *K*_D_ from this plot. As shown in Figure 3c, the binding sites of the OSMIP@silica gel for OS in the plot were favorably linearized into two segments. It was suggested that there were probably higher- and lower-affinity sites in the OSMIP@silica gel. According to the linear regression formulas, the binding constants of *K*_D1_ = 0.8516 mL·g^−1^ and *Q*_max1_ = 1203.54 nmol·g^−1^ for the lower-affinity site, and *K*_D2_ = 4.5310 mL·g^−1^ and *Q*_max2_ = 3378.25 nmol·g^−1^ for the higher-affinity site, could be obtained. As shown in Figure 3d, the binding sites of the NIP@silica gel for OS in the plot were favorably linearized into one segment. It was suggested that there was probably one kind of affinity site within the NIP@silica gel. According to the linear regression formula, the binding constant of *K*_D_ = 1.5074 mL·g^−1^ and *Q*_max_ = 902.95 nmol·g^−1^ could be obtained.

### 2.3. Specific Affinity of the OSMIP@silica Gel LC Column

**Figure 4:**
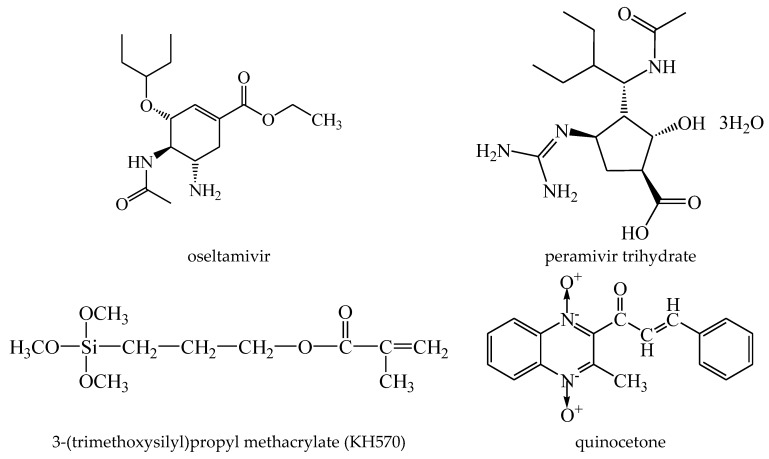
Chemical structures of the related compounds.

To develop a biological replacement method of screening for antiviral drugs, the MIP was prepared with the influenza virus neuraminidase inhibitor OS to simulate the activity center of neuraminidase. Hence, it is necessary for the specific affinity of the LC column packed with OSMIP@silica gel or NIP@silica gel to be verified. The present study showed that quinocetone (Figure 4) eluted at the same time as other impurities under the same chromatographic conditions in the OSMIP@silica gel and NIP@silica gel columns. So, quinocetone was used as the void marker.

Recognition capability of the OSMIP@silica gel column was assessed by the capacity factor (*k*′) and the imprinting factor (*IF*) [24]. The capacity factor and the imprinting factor can be calculated from the Equations (3) and (4), respectively,

(2) $$k\prime =\frac{t_{R}-t_{0}}{t_{0}}$$

(3) $$IF=\frac{k\prime_{MIP}}{k\prime_{NIP}}$$

where *t_R_* (min) is the retention time of the OS, *t*_0_ (min) is the time to elute the void marker quinocetone, and the *k*′*_MIP_* or the *k*′*_NIP_* are the capacity factors of OS from the OSMIP@silica gel column or NIP@silica gel column. The results showed that *k*′*_MIP_*, *k*′*_NIP_* and *IF* of OS were 13.52, 8.21 and 1.65, respectively. It indicated that the obtained OSMIP@silica gel column had certain recognition ability for template OS.

**Figure 5:**
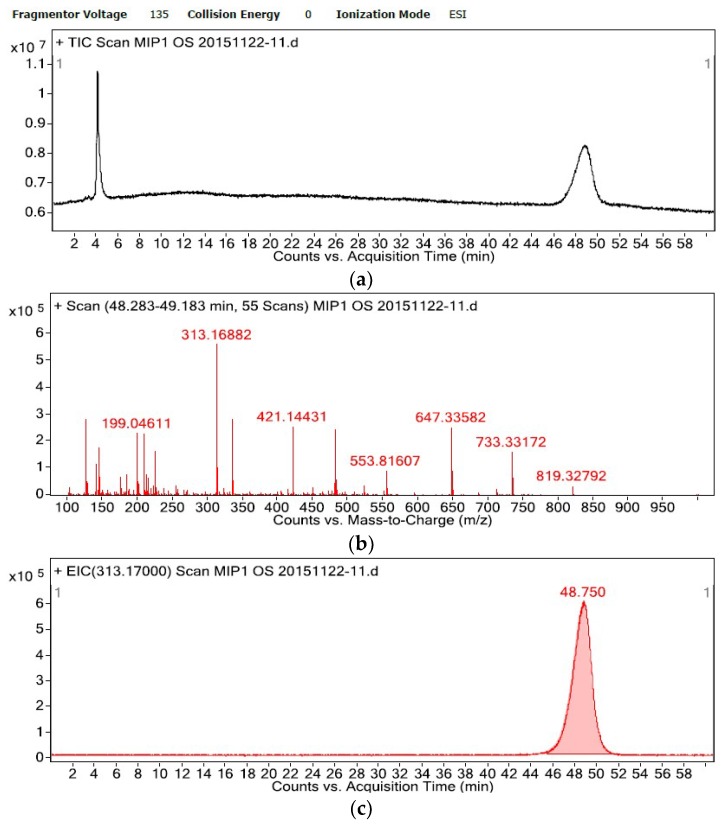
Spectra of OS standard solution on the OSMIP@silica gel column (**a**) TIC, (**b**) MS spectrum from 48 to 49 min, (**c**) EIC of *m*/*z* 313.17).

**Figure 6:**
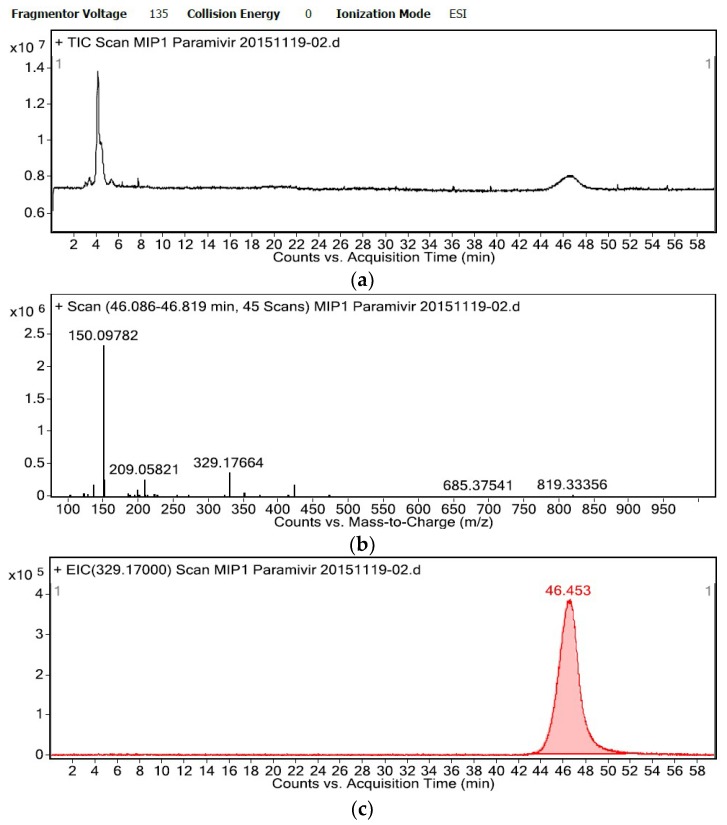
Spectra of peramivir standard solution on the OSMIP@silica gel column (**a**) TIC, (**b**) MS spectrum from 46 to 47 min, (**c**) EIC of *m*/*z* 329.17).

Peramivir, which is an influenza virus neuraminidase inhibitor and a structural analogue of OS (Figure 4), has been used to treat influenza, as has OS [25,26]. Hence, peramivir was employed to evaluate the affinity and selectivity of the OSMIP@silica gel column. Total-ion chromatograms (TICs) and extracted-ion chromatograms (EICs) of the template OS (50 μg·mL^−1^) and peramivir (40 μg·mL^−1^) are shown in Figure 5 and Figure 6, respectively, with the flow rate of mobile phase 0.4 mL·min^−1^. It is shown that the chromatographic performances, such as peak width, peak pattern and symmetry, as well as running time of OS and peramivir in the OSMIP@silica gel column have been improved significantly from the previous study [8,9].

Porous silica gel microspheres with good mechanical strength as the solid supporter of the OSMIP were considered as the main reason of chromatographic performance improvement. OSMIP@silica gel spherical particles with diameters between 6 and 8 μm are much more homogeneous than OSMIP amorphous particles with diameters between 45 and 60 μm. Therefore, the packed pressure of the OSMIP@silica gel, 400 bar (5800 psi) in the present report, is much higher than the corresponding 2000 psi of the previous study [8], and the mobile phase flow rate could be increased to 0.4 mL min^−1^. The main disadvantages of bulk MIP amorphous particles are poor accessibility to the target molecules (because of the large material thickness), slow mass transfer and problems with template removal. Molecular imprinting in porous silica gel microsphere surfaces exhibits several conspicuous advantages for chromatographic performance improvement due to recognition sites embedded on the surface, as well as structural rigidities and thermal stabilities [27,28].

The retention time of the right peak around 48.5 min in Figure 5a mainly contained OS, which is shown in Figure 5b. The ions at *m*/*z* 313.17, 335.17 and 647.34 are [M + H]^+^, [M + Na]^+^ and [2M + Na]^+^ of OS, respectively. In a similar manner, the retention time of the right peak around 46.5 min in Figure 6a mainly contained peramivir, which is shown in Figure 6b. The ions at *m*/*z* 329.18 and 351.16 are [M + H]^+^ and [M + Na]^+^ of peramivir, respectively. The ions at *m*/*z* 143.93, 209.06, 225.09, 481.76 and 553.82 in Figure 5b, as well as 150.10 and 209.06 in Figure 6b, are common ESI^+^ background ions. The remaining ions at *m*/*z* 127.00, 199.05, 421.14, 733.33 and 819.33 in Figure 5b and 136.08, 199.05, 421.15, 685.38 and 819.33 in Figure 6b are other unknown background ions.

Most of all, the retention time of peramivir on the OSMIP@silica gel column of 46.453 min (Figure 6c) was similar to the template OS of 48.750 min (Figure 5c) under the same chromatographic conditions. As we know, the retention time reflects the interaction strength between analytes and the chromatographic stationary phase. The present results indicated that the interaction between peramivir and the OSMIP@silica gel was similar to OS and the obtained particles. In agreement with the predictions, the OSMIP@silica gel had similar affinity and selectivity to the template analogue. The interaction between template OS and the OSMIP@silica gel is derived from the specific adsorption, which originates from the affinity between OS and the specific cavities of the OSMIP@silica gel. As a structural analogue, the result showed that peramivir also had a similar interaction with the OSMIP@silica gel. This indicated that peramivir would have a similar interaction to the specific cavities of OSMIP, which were generated in the imprinting processing of OSMIP. This also confirmed that the cavities of OSMIP could be used to simulate the activity center of influenza virus neuraminidase to some extent.

MIPs have two important characteristics: steric memory (size and shape) and chemical memory (spatial arrangement of the complementary functionality). They are used for separating templates or their analogues from the matrix, trapping different types of active compounds from the herbs or fermentation broth, and recognizing the inhibitors according to their bioactivities. The previous report showed that the prepared OSMIP column had similar affinity and selectivity to another compound, matrine, which had activity to inhibit influenza virus in vitro [9]. Therefore, the OSMIP@silica gel column can be also employed in follow-up studies to search for more active OS analogues from traditional Chinese herbs. Moreover, the nonbiological method for screening active components from complex matrixes by coupling an LC column with MIPs might be promoted through the present and following studies.

## 3. Experimental

### 3.1. Reagents and Solvents

Oseltamivir phosphate (purity ≥98.5%) was purchased from Hubei Shengbolai Biological Technology Co. Ltd. (Wuhan, Hubei, China). To obtain OS, oseltamivir phosphate was neutralized and extracted using sodium hydroxide and ethyl acetate, washed by saturated sodium chloride solution and dried by anhydrous sodium sulfate, and then the extract was vacuum dried at 50 °C. Peramivir trihydrate (purity of peramivir ≥85.5%) was purchased from the National Institutes for Food and Drug Control (Beijing, China). Quinocetone (recrystallized, purity 99.5% with RP–HPLC) was prepared in the Key Lab of New Animal Drug Project of Gansu Province, Key Lab of Veterinary Pharmaceutical Development of Agricultural Ministry, Lanzhou Institute of Husbandry and Pharmaceutical Sciences of CAAS. The porous silica gel microspheres (diameter 5 μm, pore volume 0.70 cm^3^·g^−1^, pore size 70 Å, surface area 400 m^2^·g^−1^) were obtained from the Lanzhou Institute of Chemical Physics of Chinese Academy of Sciences (Lanzhou, Gansu, China). Figure 4 shows the chemical structures of the related compounds in the present study.

4-Vinylpyridine (4-VP) and ethylene glycol dimethacrylate (EGDMA) were bought from Alfa Aesar (China) Chemical, Co. Ltd. (Shanghai, China). Before use, 4-VP and EGDMA were extracted with sodium hydroxide brine and dried over anhydrous sodium sulfate. Acrylamide (AA) and 3-(trimethoxysilyl)propyl methacrylate (KH570) were purchased from Sigma-Aldrich Co. (St. Louis, MO, USA). Azobisisobutyronitrile (AIBN) was purchased from Tianjin Guangfu Fine Chemical Research Institute (Tianjin, China), and recrystallized from alcohol. Toluene was purchased from Sinopharm Chemical Reagent Co., Ltd. (Shanghai, China), and refluxed with calcium hydride for one hour to obtain distilled anhydrous toluene.

Methanol (MeOH) and acetonitrile (MeCN) were purchased from Fisher Scientific (Geel, Belgium) in LC/MS grade. Formic acid was MS grade from TCI (Tokyo, Japan). MeOH, glacial acetic acid, triethylamine and hydrochloric acid in analytical-reagent grade were purchased from Sinopharm Chemical Reagent Co., Ltd. (Shanghai, China), and did not need any further purification. Deionized water (18 MΩ) was prepared with a Direct-Q^®^3 system (Millipore, MA, USA).

### 3.2. Standard Solutions

Primary OS stock solution (1 μmol·mL^−1^) was prepared in MeCN, stored at −20 °C, and was stable for 1 month (data not shown). Appropriate dilutions of OS were made in MeCN to produce working-stock solutions with appropriate concentrations on the day of experiment, and these stocks were used to perform static sorption experiments and generate the calibration curves.

### 3.3. Equipment

OS quantitative determination was performed by LC–MS/MS, which included a 1200 HPLC system with an Eclipse Plus C_18_ column (3.0 mm × 100 mm, 1.8 μm) and 6410A triple-quadrupole (QQQ) mass spectrometer with an ESI source interface operated in the positive-ion scan mode (Agilent Technologies, Palo Alto, CA, USA). Mobile phase was MeCN–H_2_O–formic acid (75:25:0.1) with flow rate of 0.4 mL min^−1^. Column temperature was 40 °C, and the injection volume was 1 μL. Parameters of the ion source in ESI^+^ and multiple-reaction monitoring were followed and optimized according to the previous research [8]. 

Heating magnetic stirrer (MR Hei-Tec, Heidolph, Germany), rotary evaporators (R215, BÜCHI, Flawil, Switzerland) and incubator shaker (Crystal Technology & Industries, Inc., Dallas, TX, USA) were also used in this research.

### 3.4. Preparation of MIP

#### 3.4.1. KH570@silica Gel [19]

10 g silica gel was reacted with 200 mL hydrochloric acid (1→2) under stirring for 24 h to remove the impurity. The silica gel was filtered by 0.22 μm filter membrane, and washed with water until the elution was neutral. Silica gel was dried at 40 °C overnight and then activated at 140 °C for 4 h.

10 g activated silica gel and 200 mL anhydrous toluene were added into the three-necked flask, 10 mL KH570 and 1 mL triethylamine were added dropwise into flask in sequence under stirring. The mixture was purged and protected with nitrogen. The derivatization was proceeded in an oil bath at 110 °C reflux for 24 h under stirring. The mixture was filtered through 0.22 μm filter, then KH570@silica gel was washed with toluene and MeOH in sequence. The silica gel was dried at 60 °C overnight and stored in dryer at ambient temperature until the following polymerization.

#### 3.4.2. OSMIP@silica Gel

OSMIP@silica gel was synthesized through the following procedure. 0.1 mmol OS (template) and 0.15 mmol AA (functional monomer) were dissolved in 30 mL anhydrous toluene (porogen) in a 250-mL three-necked flask, and 0.35 mmol 4-VP (functional monomer) was added dropwise into flask under stirring. The flask was placed in an ultrasonic water bath for 30 min, and then stirred for 3 h. Next, 5.0 mmol EGDMA (crosslinker) and 15 mg AIBN (initiator) were added into the solution under stirring. Then, 1 g KH570 modified silica gel was added into flask and stirred for 30 min at room temperature [8,17]. The mixture was purged and protected with dry argon. The polymerization proceeded in an oil bath at 50 °C for 6 h, 60 °C for 12 h and 85 °C for 6 h under stirring [18].

The nonimprinted polymer (NIP) was prepared in exactly the same way as control, except that the template molecule was absent in the polymerization stage.

The mixture was filtered through 0.22 μm filter membrane, and then OSMIP@silica gel was washed with toluene and MeOH in sequence. The template molecule OS was eluted with MeOH–acetic acid (9:1) through repeated stirring and refluxing until OS could not be detected by LC–MS/MS. The fine particles were removed by repeated sedimentation in MeOH.

### 3.5. Characterization

Fourier-transform infrared spectroscopy (FT–IR) was employed to characterize the activated silica gel, KH570@silica gel, OSMIP@silica gel and NIP@silica gel. Scanning electron microscopy (SEM) (JSM-6701F, JEOL, Tokyo, Japan) was used for morphological studies of the OSMIP@silica gel, NIP@silica gel and KH570@silica gel. The specific surface area (Brunauer, Emmett and Teller, BET) analysis and porosity (Barrett-Joyner-Halenda, BJH) measurement of the OSMIP@silica gel and NIP@silica gel were carried out at the engineering center of Lanzhou Institute of Chemical Physics of the Chinese Academy of Sciences.

### 3.6. Adsorption Experiments

Kinetic adsorption experiments were investigated by adding 20 mg of the OSMIP@silica gel or NIP@silica gel into OS MeCN solutions (5.0 mL, 5.0 nmol·mL^−1^). The mixture was shaken at a range of time from 10 to 300 min at 35 °C (120 rpm). After shaking, the samples were separated by 0.22 μm filter. The equilibrium concentration of OS in the filtered solutions was measured by LC–MS/MS [8].

Static adsorption experiments were carried out by adding 20 mg of the OSMIP@silica gel or NIP@silica gel into OS MeCN solutions (5 mL) at various initial concentrations (0.5–20.0 nmol·mL^−1^). After shaking for equilibrium time at 35 °C for 5 h (120 rpm), the samples were separated by 0.22 μm filter membrane. The equilibrium concentration of OS in the filter solutions was measured by LC–MS/MS, too. The adsorption amounts (*Q*, nmol·g^−1^) of the OSMIP@silica gel and NIP@silica gel to OS were calculated according to the Equation (1),

(4) $$Q=(C_{I}-C_{e})\times \nu ÷m$$

where *C_i_* (nmol·mL^−1^), *C_e_* (nmol·mL^−1^), *m* (g) and *v* (mL) are the initial OS concentration before adsorption, the final OS concentration in the filter liquor after adsorption, the mass of polymers and the volume of OS MeCN solution, respectively.

### 3.7. Specific Affinity of the OSMIP@silica Gel LC Column

The OSMIP@silica gel and NIP@silica gel particles were wet-packed into stainless-steel columns (150 mm × 4.6 mm i.d.) with isopropanol under pressure of 400 bar. The column was washed by LC–MS online with isopropanol, MeOH–MeCN–formic acid (60:40:0.05) until a stable baseline indicating removal of the template molecules. The specific affinity of the OSMIP@silica gel or NIP@silica gel LC column for OS and other compounds were investigated by LC–MS online.

The temperature of column was 40 °C and sample injection volume was 1 μL. The mobile phase consisted of MeOH–MeCN–formic acid (60:40:0.05). An Agilent 1290 LC tandem 6530 Q-TOF (Agilent Technologies, Palo Alto, CA, USA) with an ESI source interface operated in the positive-ion scan mode was used for the LC–MS analysis. The capillary voltage of the MS was 4000 V, the gas temperature was 350 °C, the gas flow rate was 10 L min^−1^, and the nebulizer pressure was 35 psi. Data was collected in centroid mode from 50 to 1000 *m*/*z*.

## 4. Conclusions

In this report, OSMIP@silica gel was synthesized using a surface molecular imprinting technology. The obtained particles were characterized through FT–IR, SEM, specific surface area analysis and porosity measurements. The adsorption quantity of the OSMIP@silica gel for OS was more than that for the NIP@silica gel, and the OSMIP@silica gel had two kinds of affinity sites for OS compared to one kind of affinity site in the NIP@silica gel. The imprinting factor (*IF*) of the OSMIP@silica gel column for OS was 1.64. The chromatographic performance of the OSMIP@silica gel column had significantly improved compared to previous research. What is more, the OSMIP@silica gel column showed good affinity and selectivity for template OS and another neuraminidase inhibitor, peramivir, but not for quinocetone. These results indicated that the prepared OSMIP@silica gel column could be employed in following studies to search for more affinitive compounds with bioactivity against neuraminidase from complex matrixes.

## Figures and Tables

**Table 1:** Diameter of particles, Brunauer, Emmett and Teller (BET) surface area, average pore diameter and pore volume of silica gel, OSMIP@silica gel and its corresponding NIP@silica gel (*n* = 3).

	Diameter of Particles (μm)	BET Surface Area (m^2^·g^−1^)	Average Pore Diameter (Å)	Pore Volume (cm^3^·g^−1^)
Silica gel	5	400	70	0.70
OSMIP@silica gel	6~8	244.13 ± 47.94	47.69 ± 5.57	0.29 ± 0.04
NIP@silica gel	6~8	276.29 ± 55.29	46.22 ± 4.67	0.31 ± 0.03
